# Sources and Resources‘The People’s Chemists’: The Walgreens Boots Alliance Archive

**DOI:** 10.1093/shm/hky021

**Published:** 2018-04-26

**Authors:** Anna Greenwood, Hilary Ingram

**Affiliations:** 1 Department of History, University of Nottingham, Room A5a Lenton Grove, University Park, Nottingham, UK; 2 School of Government and International Affairs, University of Durham, Al-Qasimi Building, Elvet Hill Road, Durham, UK

**Keywords:** boots, archives, British pharmacy, pharmaceutical retail

## Abstract

This article explores the historic records of the Walgreens Boots Alliance (WBA) Archive, a repository of over 500,000 items chronicling the over 165-year history of Britain’s most famous pharmaceutical retailer. It introduces some of the diverse materials present in the collection that would be of interest to social historians of medicine and pharmacy, particularly highlighting resources pertinent to understanding developments in: the formation of pharmaceutical identity; medical advertising; the internationalisation of pharmaceutical and medical retailing; product research and development; and employee welfare. In the light of the recent launch of the first phase of the Boots online catalogue, the article demonstrates ways in which these records could be utilised to better understand the wider social, cultural and political dynamics at play in the development of medicine, health care and pharmacy in Britain over the long twentieth century.

The social history of medicine is a fruitful means of investigating the lives of people in relation to their bodies and wider societal structures. Traditionally the sub-discipline relies on a combination of clinical records, doctor and patient diaries, biographies, official documentation and oral testimony. It is fair to say that company archives have not been particularly popular in most social medical historians’ archival explorations, although there have been some excellent exceptions to this general trend. Recent work by Jane Hand, for example, utilising the Unilever archive, or work by pharmaceutical historians such as Stuart Anderson, Viviane Quirke and others, informing research on nineteenth- and twentieth-century pharmaceutical developments, international competition and overseas production.^1^ Still, company archives risk being easily overlooked by social medical historians, as these resources can become pigeonholed as too self-serving of the businesses they represent, difficult to access, or thought to be too dry in terms of their holdings, hosting boxes of lacklustre accounting minutiae and turgid annual reports, rather than rich social and cultural detail.

The Walgreens Boots Alliance Archive (WBA)^2^ challenges these preconceptions and provides a unique window into the social and cultural history of medicine and pharmacy, as well as unusual insights into the commercial development of medicine and pharmacy over the nineteenth and twentieth centuries. While the archive has been in existence at the Boots main Nottingham site since the 1990s, the recent launch, in May 2017, of phase one of the Wellcome-funded WBA online catalogue provides an opportune moment to introduce this exciting resource to social medical historians.^3^ At the point of writing, 15,000 records have been added to the online catalogue, with a further 10,000 records expected in 2018. This digital cataloguing is combined with a systemic in-house conservation programme, preserving the collection—estimated to be over half a million items in total—for future generations. Although the online resource does not yet capture the entire collection, this new digital catalogue nevertheless represents an impressive sampling of the repository’s holdings: including company reports, photographs, letters, advertisements and staff magazines. Hopefully, this new interface will entice interested researchers to visit the site in person.^4^ Once they are there they will be able to additionally explore the archive’s wonderful, and even less known about, collection of material culture—from bedpans, to medicine bottles, from lipsticks to medical packaging as well as its significant film collection, providing insights into staff training initiatives, celebratory events as well as advertising campaigns.

Given the prominence of Boots as an iconic brand on every British high street, the enhanced accessibility of this collection will be a great boon to historians across a range of disciplines. To date most attention given to the archive has come from within business schools—Graeme Currie, Professor of Public Management at the Warwick Business School, has described the Boots Archive as ‘one of the largest and most comprehensive [company archives] in Europe’, while Peter Scott, Professor of Henley Business School at the University of Reading, celebrated the collection as ‘one of the most significant and multi-faceted British corporate archives’.^5^ However, outside business history, it is easy to envisage aspects of this collection also appealing to historians of labour and welfare, veterinary medicine, agriculture, marketing and design, technology, architecture and photography.

For medical historians the benefits are striking. Dating from Boots’ establishment in 1849, the primary archive spans over 165 years of dramatic change in therapies and theories.^6^ Providing such a long view, it is possible to track a variety of subjects such as: changing fashions for medication; development and stasis in marketing techniques for both pharmaceuticals and self-help remedies; the rise of company pharmacy; the growth of international pharmaceutical research and development; and the rich social and cultural histories of beauty, hygiene and home medication. Yet, despite this company’s enormous potential to act as a window into both broader societal, as well as more specific medico-pharmaceutical changes over time, the archive remains surprisingly underexplored by historians of medicine and pharmacy. Recently, the authors of this piece, Anna Greenwood and Hilary Ingram, have started a research project exploring the international history of the company, but there are many other areas of medical historical interest that could be explored through the multiple entry points offered by this collection.

The types of sources available are diverse, but a few deserve headlining as offering considerable potential. For example, the wonderfully rich staff magazine, *The Bee* (which ran from 1921–1939; 1947–1969) deserves special mention, being both an entertaining and colourful read.^7^*The Bee* is full of striking visuals, company updates, gossip and staff news—each issue packed with glossy photographs and articles spanning the frivolous to the serious. Although this in-house magazine was produced for internal consumption by Boots’ retail staff, and clearly acted as soft internal propaganda for the firm, the magazine nevertheless reveals much about changing health and beauty preoccupations over the twentieth century. Its contents could be used to shed light on changes in staff management; the professionalisation of pharmacy; attitudes to race, class and gender within pharmacy and retail; the design of chemist stores; or changing enthusiasms for different types of medical or toiletry products.

Moving from the overtly propagandist to the private professional sphere, Boots’ large collection of prescription books offer an intimate view into what people were taking for a myriad of medical ailments.^8^ Prescription books were used by the pharmacist to record details, such as patient names, medicines requested, cost, as well as specific instructions set to the patient for each prescription dispensed. Both regional and national trends could be gleaned from these sources, which offer rare insights into pharmaceutical trends over the *longue durée*. The order books from branch chemists add a surprising global dimension to this picture of quotidian life, by showing the often international sourcing of herbs, drugs, spices and exotic substances used to make up prescriptions, and giving a rare insight into the international networks and logistics that lay behind each local cross counter transaction between customer and pharmacist. Whilst the archive holds a large collection of prescription books, dating from the 1860s to 1990s, it is important to note that, as they contain personal data, they are subject to a 100-year closure period. Perhaps surprisingly, however, even after the introduction of the NHS, Boots prescription books still registered NHS prescription entries meaning they are richer than might be assumed post 1948.

Finally, even the seemingly dry, company committee minutes, annual reports and legal records, have potential beyond merely presenting a numerical analysis of profit and loss trajectories, or wins and losses in the court room. Researchers could trace, for example, the advantages and challenges associated with large-scale health care retailing through a company that had its eyes firmly on overseas expansion. Alternatively, these sources might be used to study the increasingly involved role of women within medical retailing, pharmacy, research and company leadership. In short, using the varied sources available, researchers have an opportunity to explore the history of British retail pharmacy from a much wider angle than just from within the institutional walls of Boots. Given the company’s sheer reach (by 1933, Boots had opened its 1000th store in the UK), the collection permits scholars to better understand changing trends in the British public’s consumption of drugs, self-care products, toiletries and beauty products, while also appreciating the impactful, multifaceted role played by chain pharmacy on the British high street.^9^

In the pages that follow, after briefly reminding readers of some of the company’s key dates and areas of interest, five fruitful areas that might stimulate the imaginations of social medical historians have been highlighted as examples: the shaping of pharmaceutical identity; medical advertising; the internationalisation of pharmaceutical and medical retailing; product research and development; and employee welfare.

## Introducing Boots

For those who know little about the company, a brief historical roundup detailing the scope of Boots’ developing remit and interests can help to shed light on the range of archival material available. The bones of the rags to riches story are quite well known. John Boot (1815–60) founded the first Boots store in 1849, opening a small herbalist shop on Goose Gate in the centre of Nottingham. His original business model focused on promoting the American Thomsonian herbal medical system in the region, using his modest retail outlet as a one-stop shop for customers to buy locally sourced herbal and vegetable remedies for their health and well-being.^10^ Evidence suggests that John’s business was steady but not hugely profitable; his premature death in 1860 halted any ambitions he may, or may not, have had for expansion. His widow, Mary, managed to keep the business afloat, although profits in the early days after his death were meagre. It was through the leadership of John and Mary’s son, Jesse (1850–1931), however, that business prospects improved, launching the company’s rise to commence.

By the 1870 s, Jesse’s influence began to be keenly felt in the business. In 1874, he moved away from his father’s original focus on Thomsonian herbal medicine, and instead entered the patent medicine trade: buying products at wholesale prices and selling at competitive rates.^11^ Although not qualified as a chemist himself, Jesse had an astute business sense and quickly saw the potency of a business model that actively cut out middlemen and worked directly with the drug and product wholesalers. The result was that Boot was able to offer considerably cheaper products than his competitors, thereby encouraging customer loyalty and building a brand immediately associated with selling quality for reasonable prices.

The rise of Boots was rapid. Already by the 1880s it had moved beyond retail and was starting a manufacture and production operation for its own branded drugs and medicines.^12^ In response to this growth and diversification, the company was incorporated as Boot and Company Ltd in 1883, becoming Boots Pure Drug Company Ltd in 1888. By the early 1890s, Boots was the largest company-chemist chain in Britain, and also had established its first analytical laboratory. This side of the business was to grow particularly under the impetus of the First World War in response to national priorities to sever reliance on German pharmaceutical imports. Although Boots’ research operations were never as big as some of the laboratories of continental competitors, such as Merck and Schering in Germany, or Poulenc Frères in France, Boots understood the importance of investing in drug research and development and consequently became responsible for a number of drug patents over the course of the twentieth century.^13^

Boots’ early strength was undoubtedly due to the strong visionary personality of its Managing Director, Jesse Boot, who very much oversaw all the daily business within his health care empire and ran it as a family concern. In 1920, however, this was to change and many new American business practices and conventions entered the company as a direct result of its take-over, between 1920–33, by Louis K. Liggett, owner and director of the American United Drug Company.^14^ The reason why Jesse Boot sold to an American drug firm remains somewhat unclear. Most sources suggest that after enduring years of chronic ill health, interested in raising capital to support philanthropic pursuits and ready to scale down his responsibilities, Boot lacked confidence in his son John’s ability to take over the business.^15^ Boots continued to grow during these 13 years of American ownership, adopting new business strategies that would help secure the company’s longevity in an increasingly international market.

The period after the First World War saw definite moves towards internationalisation, with Boots consciously expanding both the purchasing and distribution sides of his business overseas. By 1919 Boots had appointed its first agent to India, thereafter quickly expanding its international network of sales agents throughout the world and systemically investigating large-scale drug manufacture in overseas colonial and commonwealth contexts. By the 1960s, Boots had become an international player of worldwide significance with a strong and distinctive brand. Within the home context it acted, alongside the National Health Service, as an icon of British health care provision: a name to which everyone could easily turn and inexpensively get everything they needed in terms of medical supplies and self-help remedies. By this time the business’s wealth was sufficient to absorb several other companies into the Boots’ fold, such as the British chain of dispensing chemists Timothy Whites & Taylors (1968) and opticians Dollond & Aitchison (2009).^16^ Most recently, Boots returned to American ownership in 2014, when Walgreens acquired the company, although the familiar name and blue and white logo still remain.

## Boots as a Shaper of Pharmaceutical Identity

One area where the archive can offer particularly rich source material is the development of pharmaceutical identity. The influence Boots had on the shaping of British retail pharmacy is an important, although complex, issue within the history of pharmacy. There can be little doubt that the company had a significant (if not always welcome) impact on the evolution of much of the closely related legislature. Examining this history through the lens of the Boots archive allows researchers to explore ways in which the company has contributed to long-standing debates tracing the historically evolving relationship between professionalism and commercialism in British pharmacy. Between 1880 and 1920 particularly, Boots was involved with numerous negotiations and battles, more often than not at loggerheads with the Royal Pharmaceutical Society (RPS), who were keen to protect the interests of independent qualified chemists from the rapid market domination represented by chain pharmacies, led by Boots.^17^

Conflicts occurred as early as 1880 by which time Boot, an unqualified chemist himself, was among the first to take advantage of a timely legal case deliberated in the House of Lords in 1880. The ruling opened up new opportunities for businesses seeking to own multiple drug stores and challenged a key tenet of the 1868 Pharmacy Act, which stipulated that only qualified pharmacists, those who completed full qualifying examinations recognised by the RPS, could own and operate a shop for ‘retailing, dispensing or compounding poisons’ in Great Britain.^18^ Boot believed that while ‘pharmaceutical chemist’ and ‘pharmacist’ referenced persons deemed professionally qualified, ‘chemist’ and ‘druggist’ were terms associated with business and should, therefore, be permitted to be used by shops and corporations invested in the pharmaceutical trade.^19^ Following the 1880 House of Lords decision, Boot successfully attached the name ‘chemists’ to his business, maintaining legality by ensuring a qualified pharmacist oversaw the pharmacy and dispensed all drugs and poisons.^20^ Boot saw power in numbers and in his work to combat repeated legislative threats advanced by the RPS, he teamed, in 1898, with other multi-shop drug chains to form the Drug Companies’ Association (DCA).^21^ In 1908, Boot and the DCA struck a deal with the RPS, thereby agreeing between them the terms of the 1908 Pharmacy Act. This agreement stipulated that each multi-shop drugstore must employ a qualified pharmacist and that all companies invested in pharmaceutical dispensing and sales were only permitted to call themselves chemist or druggist if the section of the business related to pharmaceutical provision and sale was overseen by a qualified superintendent, who held a seat on the company board of directors. The move allowed Boots to retain the title ‘chemist’ and positioned the company well against other multi-shop competitors, who had to make the necessary adaptations to comply with the new legislation.^22^

The next moment of major significance, when Boot again weighed in on legislative processes, occurred in 1911; this time, for a change, speaking with the *support* of the RPS. Working with pharmacist and politician William Samuel Glyn-Jones, Jesse Boot helped to draft an amendment to the 1911 National Insurance Bill thereby protecting the right of both independent and company pharmacists to make contracts with insurance companies to dispense to insured customers under the new Act.^23^ As such, Boots should be seen as a major player in the development of retail pharmaceutical identity over the twentieth century. Company directors saw the need to be closely involved in navigating the major structural changes that occurred in health care provision during the course of the twentieth century. Even after Jesse’s death, the archive reveals how Boots was also an active player in 1948 over how pharmacies would interface with the new demands of the National Health Service.

Similarly, in the 1950s and 1960s Boots was again leading the charge to protect the interests of company pharmacy, fighting two high profile cases against the RPS. In 1952, influenced by American models, Boots experimented with self-service, where customers could pick over the counter medicines for purchase themselves. The RPS argued that self-service provision debased the image of professional pharmacy and violated the regulation that a qualified pharmacist must oversee a medicinal purchase. The case was won in favour of Boots.^24^ Similarly, in 1965, R. C. M. Dickson, Retail Director at Boots, challenged the ability of the RPS to impose a sweeping decision mandating that new pharmacies must be clearly defined and must not sell products outside of ‘traditional’ pharmaceutical goods, such as toiletries, cosmetics and photography. Dickson, supported by Boots, argued that it was not within the mandate of the RPS to enforce restrictions on areas of business that did not directly relate to pharmacy. After a three year legal battle, the RPS lost their case and all avenues of appeal.^25^

Reflecting this little-explored history of the making of British retail pharmacy, the WBA Archive carries a wealth of abundant sources. Of particular interest is the full print run of two journals, the *Chemist and Druggist* and the *Pharmaceutical Journal*, which published frequently on the legislative challenges between company pharmacy and the RPS. New perspectives can also be garnered through an examination of company committee minutes alongside the collection of bound shareholder scrapbooks.^26^ The latter deserve special mention as they present an incredible resource of clipped newspaper articles, newspaper references, general articles and advertising, providing a unique running commentary over media responses to Boots’ growth and development during the late nineteenth and early twentieth centuries.

## Medical Advertising

From the very early years of Jesse Boot’s directorship, he invested heavily in advertising both in the local and national press, widely disseminating a potent democratic message of offering affordable medicines to ‘peer and peasant alike’.^27^ The reputation of the company was also progressively enhanced by Jesse’s keen interest in, and willingness to spend money on, the design and layout of his stores. Boots stores were characterised by their plush interiors and enticing, often quite theatrical, window displays. In short, Jesse, and later his son, John, were keen to make the buying of a bargain more than just a desirable experience; they aimed to make it a pleasurable one too. Both men recognised that a good business was built on ‘display, service, salesmanship and teamwork’ and strove to impress upon their staff the importance of polite and persuasive, rather than aggressively pushy, salesmanship.^28^ The five ‘wonder stores’ that Boots opened in five different cities during the 1920 s perhaps best exemplified the company’s commitment to providing a comfortable buying experience. These ‘unique vanguard stores’ broadened the Boots offerings far beyond medicinal and toiletry products and included libraries, relaxing salons, first-aid stations, ballrooms and—from 1928—some even included hairdressers.^29^

This heavy investment in brand building, both of the company in general, and of specific products in particular, has inadvertently allowed the WBA Archive to become a treasure trove of attractive visual materials. In addition to the Dolland & Aitchinson and Timothy Whites & Taylors collections, Boots’ own material in this department is vast. The archive houses a vibrant collection of product advertising and samples for a large selection of Boots’ branded products and provides a formidable research pathway for explorations into the histories of patent medicines, self-care products, toiletries, drugs and medicines. The collection for Boots’ most famous beauty brand, No7, is particularly rich and offers pleasing insights into the development of attitudes towards skincare and beauty. Rather surprisingly, this range was launched in 1935, during the interwar years in the midst of austerity and economic recession, with the evocative slogan ‘the modern way to loveliness’.^30^ Its history provides a fascinating window into expectations regarding women, hygiene and the outward presentation of well-being and health—a trend that perhaps is most succinctly encapsulated by the growing popularity of women using make-up to create a ‘blush’, ‘radiance’ or healthy glow.^31^ Originally just sold in 50 targeted stores, the range has now become an internationally recognised brand that still enjoys popularity today.

Closely aligned to these large material collections of advertising and promotional material is the *Merchandise Bulletin*, a fortnightly journal regularly produced between 1924 and 1939 by the Merchandise Committee for Boots retail staff. This resource, alongside subsequent journals, *Selling Goes On* (1946–67), *Sales and Selling* (1953–62) and *The Bulletin* (1946–87), delivers further levels of product detail about key showcased products, often accompanied by striking visuals. Highlighted products include those outside of the Boots brand, allowing researchers to explore product range by category, and provide detail about content and efficacy, as well as pricing and intended market.^32^ The WBA Archive holds nearly 2,000 product files focusing on Boots brand development. The information contained in these files, which run from 1921 to 1980, include prices, new launches, new packaging, formulation improvements and promotional details.^33^ Researchers of products and their marketing would also be rewarded through close examination of other staff publications, such as *The Bee*, *The Mixture* (1928–39; 1940–47), *The Beacon* (1919–39; 1947–69) and *Boots News* (1970–95; 2004–07), all of which, although *The Bee* especially, regularly carried articles, advertisements and photographs from Boots’ own pharmaceutical, agricultural, veterinary and beauty ranges in order to assist staff to increase product knowledge and sales.

Finally, for researchers interested in photography and film, the archive hosts Boots’ extensive collection of photographs, dating from the late nineteenth century, as well as film holdings starting from the 1930 s. The photographic and film collections contain materials that relate not only to medical advertising and shop design and display, but include training and conference materials, product demonstrations, and employee welfare initiatives, particularly film recordings of staff field trips, or sporting and leisure events. The range of insights available to the social medical historian is vast and could be used to good effect to provide wider insights into the socio-cultural development of attitudes to fashion, gender, marketing as well as the different expectations over medical care.

## Overseas Expansion

The archive offers an abundance of material for researchers interested in the international development of medicines and global networks of pharmaceutical exchange. Although rarely acknowledged, from the earliest stages of its development, Boots was engaged with the wider world: even at a time when ‘internationalisation’ as we conceive of it now, was far from being on the company’s official agenda. In line with the historic traditions of pharmacy, Boots sourced materia medica from across the globe, over time expanding its imports to also include raw materials necessary to aid product manufacture in Nottingham, and later expanding imports and exports to ready-made products themselves.

During the twentieth century Boots, unsurprisingly, worked most closely with colonial partners, primarily (but not exclusively) pursuing business ventures in established British colonies. In 1919, Boots secured its first sales in India, selling Boots branded products through an independent agent. In the 1920 s and 1930 s, company representatives travelled to countries such as Australia, Canada, India, Myanmar, New Zealand, Russia, Sierra Leone and the USA to explore opportunities for trade and collaboration. Several of these countries housed Boots subsidiaries by the mid-1920 s to facilitate trade. The company ventured into Africa in the 1940 s, identifying potential markets and critical research needs across the continent.^34^

Although by the 1930 s many chemist shops around the world routinely carried Boots’ lines, in terms of the development of retail, Boots’ success was relatively modest. It established its first shops in New Zealand in 1936, followed by Fiji in 1944. In these, and other, overseas ventures, the company clearly targeted British expatriates more than local populations, framing overseas expansion as a service to ‘loyal citizens of empire’.^35^ Comparatively enlightened for the time, however, Boots pledged to hire locally. Consequently, Boots became a relatively large international employer, with local staff hired for their branch offices, factories, warehouses and research laboratories.

The archive holds files associated specifically with Boots’ international wholesale business, with relevant papers in the collection dating between 1919 and 2006, as well files for each of the countries in which the company operated.^36^ Indeed, information about Boots’ international reach can be found throughout the collection within the annual reports, shareholder scrapbooks and staff magazines. *The Bee*, for example, frequently contained articles about international trade from its first publication in 1921 and regularly entertained its readers with accounts relating the visits of managers abroad, updates on new international products and announcements about new concessions and inroads negotiated overseas.

Of additional interest, the archive also has extensive holdings revealing the challenges against Boots’ international ambitions. The company faced Government inquiries in both New Zealand and Australia, as existing self-employed pharmacists feared the threat of ‘company chain pharmacy’, selling at lower prices.^37^ The copious papers related to these government enquiries are a valuable resource for researchers interested in the history of British retail pharmacy abroad as well as those looking at the broader evolving relationship between pharmacy and Empire.^38^ Barely consulted, the transcripts and testimonies from the cases against this British encroachment reveal how expert opinion was solicited across British colonies, with Pharmaceutical Associations called upon to share best practice and comparative experience from across the globe.

## Research and Development

There is much for the historian interested in drug research and development. Between the 1880 s and 1980 s, Boots Research Department grew into a dynamic and varied operation, with company scientists operating specialist divisions in Bacteriology, Antibiotics and Fermentation, Chemotherapy, Virology, Biochemistry, Drug Metabolism, Physical Chemistry, Pharmacology and Medical Sciences over this period.^39^ Despite being primarily known for its retail presence, in many areas Boots contributed to leading pharmaceutical research. It is often forgotten, for example, that Boots’ research laboratories pioneered the, now world famous, painkiller Ibuprofen (originally launched as Brufen) during the 1960s; that Boots was amongst the first British companies to be given a licence to distribute and manufacture insulin for the UK in the 1920s; or that it was the British government’s leading provider of penicillin during the Second World War. For these products, alongside many others, the WBA Archive hosts numerous records related to product research, testing, marketing and production.^40^ Furthermore, a focus on any of these (or other) drugs highlights, not only the often turbulent life cycle of pharmaceutical products from conception to shop shelves, but also offer insights into a dramatically changing historical landscape regarding expectations regarding pain control, as well as changes in the management of chronic diseases.

Drug research and development files also reveal the way research was closely related to political agendas, promoting British business as a leader in tending to the health care of the nation. In 1945, Boots directors were proud to report that they had ‘been able to supply the essential needs of the home market during the war’, thus tangibly contributing to the national war effort, through their home production of aspirin, saccharin, potassium permanganate and penicillin.^41^ The research and development files relate not only to drug development, testing and production within Boots’ own laboratories, but crucially act as signposts to wider developments across the pharmaceutical scene. There are records on research processes, product development, efficient storage as well as abundant documentation examining pricing, packaging and marketing.

The type of source material is varied, from a collection of botany specimens and hand-written formula books referencing Boots branded products dating from the late nineteenth century, to documents detailing dedicated research endeavours focusing on antibiotic resistance in the 1950 s.^42^ Information is also scattered through the numerous committee minutes, annual reports, product files as well as within the *Monthly Medical Sales Bulletin*, later known as *The Hexagon*, reporting (albeit subjectively) successes and challenges within drug research and development.

Finally, Boots’ interest in agricultural and veterinary product development should not be forgotten. Although Boots has been commissioning research into these areas since the late 1920 s, it was in 1935 that the Agricultural and Horticultural Division of the Research Department was officially opened. Boots eventually bought their own veterinary research centre, Lenton Research Station, in 1939, with further farmland purchased and laboratories established exclusively for the agricultural and veterinary market in the 1940 s. Boots focused on a wide variety of experimentation and drug trials throughout the 1950 s and 1960 s, with successes including: a much hailed treatment against red spider, the production of agricultural antiseptics, trypanosomiasis trials on cattle in East Africa, and attempts to reduce the threat of bovine tuberculosis in Britain. An Agricultural Research Station was also built in Kooree, Australia in 1968 to provide Boots with a facility to test in a semi-tropical climate. The archive houses several boxes of materials related to the lesser-known history of Boots’ involvement with agricultural and veterinary production, ranging from staff and retail sales magazines, retail advertising, operational reports submitted by each farming station as well as specific product research files. The Agricultural and Veterinary Divisions of the Research Department were closed in 1981 following a merger between Boots and Fissons Agrochemical.^43^

## Employee Welfare

From the late nineteenth century, Boots became increasingly associated with groundbreaking practices in industrial welfare and community support.^44^ Especially after his marriage to Florence Rowe in 1886, Jesse Boot invested much time in his staff: both in terms of their recreational opportunities and educational training. Under the guidance of Jesse and Florence, a whole series of staff clubs proliferated with the athletic club amongst the earliest to be founded in 1894, with other clubs and amenities quickly following, especially during the first quarter of the twentieth century. Reflecting his abiding interest in education, Boots Day Continuation School was established in 1920 ‘to mould the personal, social and working characteristics of teenage employees’, as well as a ‘Progress Club’ which delivered helpful fortnightly lectures to managers.^45^ Boot also extended his educational welfare outside of his company, most importantly donating large amounts of his land and fortune to the city’s university. Compared to other companies of the time, Boots was unusually focused on providing welfare support for his staff and it has been estimated that by 1914 Boots employed four of only 60 industrial welfare officers in existence in the UK.^46^ In terms of far reaching impact in this area, perhaps the highest profile moment in the Boots’ history of employee welfare came in 1934 when Boots became the first large-scale company to introduce the five-day working week by shutting down factory production on Saturdays and Sundays—a move which was to change the landscape of British employment history forever, ultimately stimulating parliament to amend its legal requirements over statutory working hours.^47^

The WBA Archive holds ample information relating to the Boot family and their philanthropic and employee welfare endeavours, casting light more broadly on changing priorities and provision for employees from the end of the nineteenth century until the modern day. Information contained in annual reports, staff magazines, purchasing and inventory ledgers, alongside both staff and family correspondence can be pieced together to build a fascinating picture of these far-reaching and varied welfare initiatives. In this realm Florence Boot, has been a particularly under appreciated figure, although the archive reveals the extent of her impact on company direction and purchasing, her influence on training and welfare initiatives for female employees, as well as her work in developing the Boots Booklovers Library, a lending library for subscribing customers that, at its peak, was present in 460 Boots stores from 1897–1966.^48^

## Conclusion

Although this short introduction has, necessarily, just touched the tip of the iceberg in terms of highlighting the WBA Archive’s potential, the usefulness of this resource for social medical historians is certainly without doubt. As with any company archive, researchers need to be aware that success stories and celebrations are easier to locate within the archive than company controversies and complaints. Furthermore, when these less hagiographic representations are present, they tend to be embedded within larger debates supporting company development and may not be immediately obvious to the researcher. The company magazines, for example, could be singled out for presenting an overly positive image of the company although even these highly subjective sources, if used cautiously and critically, can offer rich insights into changing social and cultural priorities over the course of Boots’ history.

With the launch of the online catalogue last May, increasing numbers of researchers will inevitably find their way to utilising this collection, which offers far more than just prosaic insights into the internal mechanisms of a large pharmaceutical chain. Holdings shed light on a broader world of medical trends, medical advertising, employee welfare, the growth of international pharmacy as well as changing attitudes relating health, gender and beauty. Corporate archives tend to be underused by social medical historians, but with imagination and insight they can become keys that help us understand the commercial side of medicine and pharmacy in shaping the health and beauty conscious world in which we all live today.

## Footnotes

1 Stuart Anderson, ed., *Making Medicines: A Brief History of Pharmacy and Pharmaceuticals* (London: Pharmaceutical Press, 2005); Roy A. Church and E. M. Tansey, *Burroughs, Wellcome & Co.: Knowledge, Trust, Profit and the Transformation of the British Pharmaceutical Industry, 1880–1940* (Lancaster: Crucible, 2007); T. A. B Corley, *Beecham’s, 1848–2000: From Pills to Pharmaceuticals* (Lancaster: Crucible, 2011); R. P. T. Davenport-Hines and Judy Slinn, *Glaxo: A History to 1962* (Cambridge: Cambridge University Press, 1992); Jane Hand, ‘Marketing Health Education: Advertising Margarine and Visualising Health in Britain from 1964–C.2000’, *Contemporary British History,* 2017, 1-24; Viviane Quirke and Judy Slinn, eds, *Perspectives on Twentieth-Century Pharmaceuticals* (Oxford: Peter Lang, 2010); Lucas Richert, *Conservatism, Consumer Choice, and the Food and Drug Administration During the Reagan Era: A Prescription for Scandal* (Lanham: Lexington Books, 2014).

2 Walgreens Boots Alliance Archive: WBA/BT/4/15/1/2, *Truth—Industrial Supplement*, 1912, 26, i–xii, at i.

3 Walgreens Boots Alliance, Inc., ‘Walgreens Boots Alliance Archive Catalogue’, <http://archives.walgreensbootsalliance.com/>, accessed 27 May 2017.

4 The archive is located at the Walgreens Boots Alliance headquarters in Nottingham, UK and is open to researchers, by appointment, Monday to Friday from 9am to 4pm. If interested in visiting, please email bootsarchives@wba.com.

5 Warwick Business School, **‘**WBS to open up Boots Archive**—**one of Europe**’**s biggest**’**, <https://www.wbs.ac.uk/news/wbs-to-open-up-boots-archive-one-of-europe-s-biggest/>, accessed 1 November 2016; Peter Scott, *The Boots Company Archives: An Academic Appraisal* (Unpublished, 2014), 58.

6 WBA records are subject to a standard 30-year closure that is applied across the business collection. Where records contain sensitive personal data, these materials are subject to a 100-year closure. Requests for materials within these closure periods would have to be reviewed and anonymised by the archives team on a case-by-case basis, if access was to be granted at all.

7 WBA/BT/27/39/2/2, *The Bee*.

8 WBA/BT/34/20/1, Prescription Books.

9 WBA/BT/27/39/2/2/114, *The Bee*, 1933, 13, Front Cover.

10 Stanley Chapman, *Jesse Boot of Boots the Chemists: A Study in Business History* (London: Hodder and Stoughton, 1974), 35.

11
*Ibid.*, 37.

12 WBA/BT/28/40/4/14, Development and Organisation of Boots Pure Drug Company Ltd., 2.

13 Jonathan Liebenau, ‘The Twentieth-Century British Pharmaceutical Industry in International Context’, in J. Liebenau, G. J. Higby and E. C. Stroud, eds, *Pill Peddlers: Essays on the History of the Pharmaceutical Industry* (Madison, WI: American Institute of the History of Pharmacy, 1990), 130; Viviane Quirke, ‘Foreign Influences, National Styles, and the Creation of a Modern Pharmaceutical Industry in Britain and France’, *Pharmacy in History*, 2010, 52, 134–47.

14 United Drug Company was renamed Rexall Drug Company in the 1940s, although stores and branded products were commonly known under the ‘Rexall’ name throughout the period of Boots ownership.

15 Paul Whysall, ‘Interwar Retail Internationalization: Boots under American Ownership’, *The International Review of Retail, Distribution and Consumer Research*, 1997, 7, 157–69, at 160–1; John Eric Greenwood, *A Cap for Boots: An Autobiography* (London: Hutchinson Benham, 1977), 10, 16.

16 WBA/BO/DA, Dollond & Aitchison; WBA/BT/TW, Timothy Whites & Taylors Ltd.

17 S. W. F. Holloway, *Royal Pharmaceutical Society of Great Britain 1841–1991: A Political and Social History* (London: The Pharmaceutical Press, 1991), 311.

18
*Ibid.*, 278; Stuart Anderson, ‘From “Bespoke” to “Off-the-Peg”: Community Pharmacists and the Retailing of Medicines in Great Britain, 1900–1970’, in Louise Hill Curth, ed., *From Physick to Pharmacology: Five Hundred Years of British Drug Retailing* (Aldershot: Ashgate, 2006), 126; Chapman, *Boots the Chemists*, 59.

19 Holloway, *Royal Pharmaceutical Society*, 241.

20
*Ibid.*, 278.

21
*Ibid.*, 299.

22
*Ibid.*, 304; Peter G. Homan, ‘The Development of Community Pharmacy’, in Stuart Anderson, ed., *Making Medicines: A Brief History of Pharmacy and Pharmaceuticals* (London: Pharmaceutical Press, 2005), 122–5.

23 Holloway, *Royal Pharmaceutical Society*, 335.

24
*Ibid.*, 372–3.

25
*Ibid*.; WBA 2978; WBA 534/4&5; WBA/BT/12/2/36/3, Pharmaceutical Society and Legislation.

26 WBA/BT/4/15/1, Shareholder Scrapbooks. Back issues of *Chemist and Druggist* have been digitised by the Wellcome Library and are available to view via Internet Archive as well as through the Library catalogue.

27 WBA/BT/4/15/1/2, *The Trader: An Illustrated Journal for Business Men*, 1910, *3*, Front Cover.

28 WBA/BT/27/39/2/2/112, ‘Business Building’, *The Bee*, 1933, 12, 284.

29 Simon Phillips, ‘“Chemists to the Nation”: House Magazines, Locality and Health at Boots the Chemists 1919–1939’, *Management and Organizational History*, 2008, 3, 239–55, at 246; WBA/BT/27/39/2/2/78, *The Bee*, 1928, *7*, Front Cover.

30 Anna Chesters, ‘A Brief History of No7’, *The Guardian*, 16 April 2012, <https://www.theguardian.com/fashion/fashion-blog/2012/apr/16/brief-history-of-no7-boots>, accessed 5 July 17.

31 Richard Hornsey, Manuscripts and Special Collections, University of Nottingham, and Walgreens Boots Alliance Archive, ‘Inspiring Beauty: No7—80 Years of Making Up the Modern Woman’, Weston Gallery, Nottingham Lakeside Arts, 15 January–17 April 2017.

32 WBA/BT/11/38/1/1, *Merchandise Bulletin* and Supplements; WBA/BT/11/38/1/2, *Selling Goes On*; WBA/BT/11/38/1/3, *The Bulletin* and Supplements; WBA/BT/11/38/1/4, *Sales and Selling*.

33 WBA/BT/9/22, Product Files; WBA/BT/11/22, Product Files.

34 WBA/BT/12/17/1/4, R. M. Dickson and E. M. Phillip, A Survey of Africa, 1949.

35 WBA/BT/NZ/4/1/4, Preliminary Negociations in New Zealand, 1935, 17.

36 WBA/BT/12, International Wholesale.

37 Gregory Haines, *‘The Grains and Threepenn’orths of Pharmacy’: Pharmacy in N.S.W., 1788–1976* (Kilmore: Lowden Publishing, 1976), 181–2; Gregory Haines, *Pharmacy in Australia: The National Experience* (North Sydney: Australian Pharmaceutical Publishing, 1988), 284–93; Reginald Edmund Combes, *Pharmacy in New Zealand: Aspects and Reminiscences* (Auckland: R. Richards, 1981), 149–58. For more on the New Zealand challenge, see Pauline Norris, ‘Changes in New Zealand Pharmacy, 1935–1945’, *Pharmacy in History*, 1995, 37, 165–75.

38 WBA/BT/2/55/1, Report of Proceedings of Inquiry into the Petitions of The Pharmaceutical Society of New Zealand, Boots Pure Drug Col. Ltd., The Wholesale Druggists Association of New Zealand, The Dunedin Retail Chemists’ Assistants’ Industrial Union of Workers; WBA/BT/AU/4/4/1/1, Australia Chemist Chain Stores Public Inquiry, Supporting Materials.

39 WBA/BT/8/51/1, A. E. Davis, Research and Development at Boots: Notes on the History of Research and Development Showing Its Evolution Alongside Manufacturing and Quality Control, 22–9.

40 For a small sampling of relevant records available at the WBA Archive, see: WBA/BT/9/22/10/233, Insulin Product Files; WBA/BT/8/17/1/1, Report of a Visit to Biochemico Ltd.; WBA/BT/ID/4/2/6, India Insulin Project; TRC, Therapeutic Research Corporation of Great Britain Ltd Papers; WBA/BT/9/45/1, Brief Description of the Production of Penicillin at the Daleside Road Factory, Nottingham; WBA/BT/11/40/4/5/19, Bridging a Gap: The Story of Achievement in the War-time Production of Penicillin; WBA/BT/9/22/10/68, Brufen Tablets Product File; WBA/BT/12/36/51, International Wholesale, Ibuprofen Information and Licence Interest.

41 WBA/BT/4/15/2/7, The Chairman’s Speech, 57th Annual General Meeting, 26 July 1945, 4.

42 WBA Y37, Botany Specimens; WBA/BT/9/23/2, Formula Books; WBA/BT/11/38/3/1, ‘Drug Resistance and the Treatment of Bacterial Infections’, *The Hexagon*, 1959, 1, 6–9.

43 WBA/BT/8/51/1, Davis, Research and Development at Boots, 35–6.

44 Simon Phillips, ‘“Fellowship in Recreation, Fellowship in Ideals”: Sport, Leisure and Culture at Boots Pure Drug Company, Nottingham C. 1883–1945’, *Midland History*, 2004, 29, 107–23; Phillips, ‘Chemists to the Nation’.

45 Simon Phillips, ‘“Character, Grit and Personality”: Continued Education, Recreation and Training at Boots Pure Drug Company 1918–45’, *History of Education*, 2003, 32, 627–43, at 627; Chapman, *Boots the Chemists*, 174.

46
*Ibid.*, 169.

47 ‘A Five-Day Week: Experiment at Boots’s Factories’, *The Times*, 28 April 1934.

48 WBA/BT/16/1, Library.

## Notes

Anna Greenwood and Hilary Ingram are social and cultural historians of Britain and the British colonial world, with a particular interest in medical history. This article results from research conducted for Greenwood’s Wellcome Seed Award, ‘Boots and the Colonial World: Imperial Networks and the Business of Empire’ (September 2016–August 2017).

